# Judicious use of critical care resources by predicting the need for routine ICU admission following esophagectomy

**DOI:** 10.1093/dote/doaf075

**Published:** 2025-09-17

**Authors:** Nikki de Mul, Lisette M Vernooij, Nynke C J van Haastregt, Eline M de Groot, Willem-Jan M Schellekens, Lennie P G Derde, Jelle P Ruurda, Olaf L Cremer

**Affiliations:** Department of Intensive Care Medicine, University Medical Center Utrecht, Heidelberglaan, Utrecht, the Netherlands; Department of Anesthesiology, University Medical Center Utrecht, Heidelberglaan, Utrecht, the Netherlands; Julius Center, University Medical Center Utrecht Universiteitsweg, Utrecht, the Netherlands; Department of Intensive Care Medicine, University Medical Center Utrecht, Heidelberglaan, Utrecht, the Netherlands; Department of Anesthesiology, University Medical Center Utrecht, Heidelberglaan, Utrecht, the Netherlands; Department of Intensive Care Medicine, University Medical Center Utrecht, Heidelberglaan, Utrecht, the Netherlands; Department of Surgical Oncology, Upper Gastro-intestinal Surgery, University Medical Center Utrecht Heidelberglaan, Utrecht, the Netherlands; Department of Anesthesiology, University Medical Center Utrecht, Heidelberglaan, Utrecht, the Netherlands; Department of Anesthesiology, Cantonal Hospital Aarau, Aarau, Switzerland; Department of Intensive Care Medicine, University Medical Center Utrecht, Heidelberglaan, Utrecht, the Netherlands; Department of Surgical Oncology, Upper Gastro-intestinal Surgery, University Medical Center Utrecht Heidelberglaan, Utrecht, the Netherlands; Department of Intensive Care Medicine, University Medical Center Utrecht, Heidelberglaan, Utrecht, the Netherlands

**Keywords:** complications, critical care medicine, epidemiology, esophagectomy, intensive care

## Abstract

With advances made in the care for esophagectomy patients, the need for routine postoperative intensive care unit (ICU) admission needs reassessment. We developed a preoperative prediction model to distinguish patients requiring routine ICU admission from those who can be cared for in a post-anesthesia care unit (PACU).

This retrospective cohort study included consecutive adults undergoing elective esophagectomy between January 2011 and June 2021 in the UMC Utrecht. Firth’s corrected multivariable logistic regression was used for model development and internal validation using bootstrapping was performed to obtain optimism-corrected performance metrics.

Among the 619 patients included, 380 (61%) received critical care support beyond the first morning following surgery: 83 (13%) were on invasive mechanical ventilation and 338 (55%) needed cardiovascular support (with 174 [28%] receiving only low-dose norepinephrine). Predictors retained in the final model included age, diabetes mellitus, hemoglobin level, kidney function, forced expiratory volume in 1 second, tumor stadium, type of neoadjuvant therapy and surgical approach. Discrimination was acceptable (adjusted *c*-statistic 0.67, 95% CI 0.62–0.71) with good calibration (*O*:*E* ratio 1.00). Using the model, approximately 50% of ICU beds could be conserved, at the cost of misallocating 22% of patients to a PACU (with only 12% of PACU-allocated patients requiring mechanical ventilation). Between one- and two-thirds of elective esophagectomy patients do not need routine ICU admission, depending on whether hemodynamic support can be provided in another high-dependency unit. Our model can help rationalize perioperative patient allocation and reduce ICU bed claims by roughly half.

## INTRODUCTION

Surgical treatment of esophageal cancer has advanced significantly over the past decade. This includes the use of minimally invasive surgical methods, alternative anastomotic techniques (such as cervical vs intrathoracic, or hand-sewn vs stapled anastomosis) and enhanced recovery protocols after esophagectomy.^1^ Despite these advances, complications following esophagectomy remain frequent, with an estimated overall risk of complications in the Dutch Upper Gastrointestinal Cancer Audit (DUCA) of 59%, the most common types being pneumonia (15%), atrial dysrhythmias (15%) and anastomotic leakage (11%).^2^ Most of these complications occur between 3 and 10 days after surgery.^3^

In the past, patients undergoing total esophageal resection with gastric pull-up were routinely admitted to the intensive care unit (ICU) postoperatively and mechanically ventilated overnight to prevent aspiration, protect the airway and allow for adequate pain management during the immediate postoperative period.^4^ With the introduction of enhanced recovery protocols, patients are now commonly extubated in the operating room. This was not only shown to be feasible and safe, but has also been associated with reduced morbidity^4^^,^^5^ and ICU length of stay.^6^ With these changes in postoperative care, the need for routine ICU admission following esophagectomy has been questioned.^7^ Indeed, the current guidelines for perioperative care in esophagectomy patients therefore advise to individualize postoperative management of patients after esophagectomy and consider the post-anesthesia care unit (PACU) or another high-dependency unit (HDU) as a safe alternative for lower risk patients.^7^ Bed capacity is scarce and admitting patients to a PACU instead of ICU might lead to a significant cost reduction.^7^ Therefore, we evaluated whether routine ICU admission following esophagectomy is still necessary. To optimize judicious use of critical care resources, we developed a preoperative prediction model to distinguish esophagectomy patients requiring ICU admission from those who may be cared for in a PACU.

## METHODS

This retrospective cohort study was conducted at the University Medical Center Utrecht, a tertiary academic hospital and designated major trauma center in the Netherlands. All adults undergoing elective esophagectomy between January 2011 and June 2021 were included. The preoperative work-up involved evaluation at a preanesthesia clinic, including spirometry as per European Respiratory Society and American Thoracic Society (ERS/ATS) guidelines,^8^ and optimization of modifiable risk factors such as anemia, hypertension, pulmonary disease, malnutrition and diabetes. Thoracic epidural analgesia was used as standard, except for 10 patients who received paravertebral blocks as part of (or preparation for) the PEPMEN trial (Netherlands Trial Registry NL8037). Esophageal resection was performed either via a transthoracic or transhiatal approach and always included a gastric pull-up procedure. Subsequently, patients were routinely admitted to the ICU for postoperative management. Subjects enrolled between 2011 and 2016 had been extubated at the discretion of the treating physician, whereas from 2016 onwards an Enhanced Recovery After Surgery (ERAS) protocol was implemented that prescribed early extubation in the operating room. According to the Institutional Review Board (IRB) of the University Medical Center Utrecht, this study was not subject to the Medical Research Involving Human Subjects Act (IRB number 21/488). Data were used under the no-objection policy of the General Data Protection Regulation (GDPR).

We combined data from a prospectively maintained institutional surgical database, the Molecular Diagnosis and Risk Stratification of Sepsis (MARS) biorepository (NCT01905033), and clinical data extracted from electronic health records to develop a prediction model that could be used for the preoperative decision to allocate either a postoperative PACU or ICU bed to patients undergoing elective esophagectomy. In addition, we developed an extended model that (also) incorporated intraoperative information to explore whether this information would significantly improve model predictions. Reporting of our study is in accordance with the Transparent Reporting of a Multivariable Prediction Model for Individual Prognosis or Diagnosis (TRIPOD) guidelines.^9^

### Outcome definition

We considered postoperative allocation to an ICU bed appropriate if the patient’s condition on the first day after surgery (i.e. 10–24 hours postoperatively) mandated the continued use of respiratory and/or hemodynamic support measures that are typically reserved for a critical care unit. These measures included (i) use of invasive or noninvasive mechanical ventilation or high-flow nasal oxygen therapy and/or (ii) continuous infusion of norepinephrine, phenylephrine, dobutamine or milrinone at any dose. Of note, in a post hoc sensitivity analysis we used a stricter definition of vasopressor support, which mandated a norepinephrine continuous infusion rate of ≥0.1 μg/kg/min (or equivalent), as lower doses of vasopressor support are commonly given outside the ICU in clinical practice (e.g. in HDUs). We considered postoperative allocation to a PACU bed appropriate if neither criterion 1 nor 2 was met, indicating respiratory and hemodynamic stability.

### Candidate predictors

Candidate predictor variables were selected a priori, based on clinical experience and a comprehensive literature review of published risk factors for postoperative complications (Supplementary Table 1).^10–15^ Comorbidity was defined according to the Netherlands Intensive Care Evaluation (NICE) registry criteria.^16^ Cardiovascular comorbidity was a composite of severe coronary artery disease, myocardial infarction, heart failure, peripheral vascular disease and cerebrovascular disease in the medical history.

Univariable analyses were used to explore nonlinear relationships of continuous variables with the outcome using restricted cubic splines. Only the preoperative hemoglobin level clearly showed a nonlinear relationship to the outcome and was categorized with three levels: low (Hb < 7.5 mmol/L), intermediate (Hb 7.5–8.5 mmol/L) and high (Hb > 8.5 mmol/L).

Missing values were observed in six variables (range 1%–9% missingness, supplementary materials). We assumed that missingness was at random and performed multiple imputation by a chained equation to generate 10 imputed datasets.^17^

### Statistical analysis

We developed the prediction model using logistic regression analysis. We used the criteria proposed by Riley *et al.* to determine that our approach would allow for the modeling of up to 25 degrees of freedom (Supplementary Table 2).^18^ Therefore, we first fitted a fully saturated model that included all 16 preoperative candidate predictors (spending 18 degrees of freedom) and subsequently used backward selection of covariates using Akaike’s information criterion.^19^

For model fitting, we used Firth’s penalized logistic regression, which is the preferred method for model development in smaller datasets when the event per candidate predictor ratio approaches 10:1. The Firth correction shrinks the intercept and all regression coefficients individually during the initial model development process.^20^ This reduces the risk of overfitting (i.e. improves external validity) and it provides more stable results compared to other shrinkage techniques.^21^ We performed this modeling approach over each imputed dataset and pooled the results using Rubin’s rules. The final model was established using a majority vote (i.e. we selected those predictors that had been retained in ≥50% of the imputed datasets) followed by a Wald test until no further variables could be removed.

We evaluated model performance by assessing discriminatory ability, calibration and overall predictive accuracy. Discrimination was evaluated using the *c*-statistic and visualized with the area under the receiver operator curve (AUROC). Calibration was evaluated using the observed to predicted (*O*:*E*) ratio, the calibration intercept and the calibration slope.^22^ Accuracy was expressed by the (rescaled) Brier score. Internal validation was performed by bootstrapping (using 500 iterations) to correct for optimism in the reported model performance estimates.^23^ To determine the optimal decision threshold for routine ICU admission, we assessed several predicted probability cutoffs ranging from 0.55 to 0.80.

Finally, following development of the preoperative model, we studied whether it could be further improved by additionally incorporating intraoperative predictors that are known at the conclusion of surgery. The goal of this sensitivity analysis was to assess whether the need for an ICU bed predominantly depends on preoperative information or is mostly determined by intraoperative events. For development of this early postoperative (extended) model, we used a similar analytical approach to that described above.

Continuous variables were compared between groups using either a two-samples *t*-test or Mann–Whitney *U* test, whichever was appropriate. Differences in categorical variables were compared using the chi-square test. All the analyses were performed in R version 4.0.3 (R Foundation for Statistical Computing, 2020) using packages ‘mice’, ‘pmsampsize’ and ‘logistf’.

## RESULTS

All 619 adult patients who had undergone an elective esophagectomy between January 2011 and June 2021 were analyzed. For logistical reasons, 13 patients had been admitted to the PACU, whereas all the others were electively admitted to the ICU following surgery. In total, 380 patients (61%) met the criteria for routine ICU admission, whereas admission to a PACU with subsequent transfer to a regular ward the next morning would have been possible for 239 (39%) patients. For the 380 patients for whom ICU care proved necessary after surgery, respiratory and/or hemodynamic support included the continued use of (a combination of) invasive mechanical ventilation in 83 (13%), noninvasive ventilation in 28 (5%), inotropes and/or high-dose vasopressors in 134 (22%) and low-dose vasopressors in 204 (33%) subjects. Of note, 174 (28%) patients received only low-dose vasopressors without respiratory support. This proportion increased after introduction of an ERAS protocol in 2016 (from 20% in 2011–2015 to 37% in 2018–2021), as did the number of on-table extubations (from 47% to 91%, respectively).

Patients who required postoperative allocation to an ICU bed were older and had more cardiovascular comorbidities then remaining subjects (Table 1). They also frequently had a middle or upper esophageal tumor, received neoadjuvant chemoradiation, or underwent an open and/or transthoracic procedure. Finally, they suffered more blood loss (>1000 mL) and consequently received more transfusions. On-table extubation rates and ICU readmission rates were similar for both groups (Table 1).

**Table 1:** Characteristics of esophagectomy patients with and without postoperative ICU dependency

	ICU dependency (*n* = 380)	PACU eligible (*n* = 239)	*P*-value
**Preoperative patient characteristics**			
Age (years)	68 [62–73]	65 [56–70]	<0.001
Sex at birth (M), *n* (%)	271 (71)	185 (77)	0.110
BMI (kg/m^2^)	25 [22–27]	25 [23–28]	0.230
ASA score, *n* (%) 1 + 2 3 + 4	261 (70) 114 (30)	187 (79) 51 (21)	0.020
COPD, *n* (%)	54 (14)	31 (13)	0.750
Cardiovascular comorbidity^*^, *n* (%)	87 (23)	37 (16)	0.030
Diabetes mellitus, *n* (%)	47 (12)	36 (15)	0.400
Hypertension, *n* (%)	134 (35)	78 (33)	0.560
Smoking history, *n* (%)	90 (24)	45 (19)	0.190
**Preoperative biomarkers**			
Hemoglobin (mmol/L)	7.8 [7.2–8.6]	8.1 [7.4–8.6]	0.040
Kidney function, *n* (%) Good (eGFR > 60 mL/minute) Dysfunction (eGFR ≤ 60 mL/minute)	325 (91) 34 (9)	218 (95) 12 (5)	0.100
FEV1 (%pred)	98 [83–109]	100 [88–113]	0.030
Tiffenau index (%pred)	97 [87–104]	98 [90–104]	0.180
**Surgical characteristics**			
Tumor location, *n* (%) GE junctional Lower Middle Upper	46 (12) 240 (64) 65 (17) 24 (6)	40 (17) 156 (68) 29 (13) 5 (2)	0.020
Type of neoadjuvant therapy, *n* (%) None Chemo-/radiotherapy alone Chemoradiation	58 (15) 33 (9) 288 (76)	31 (13) 57 (24) 148 (63)	<0.001
Type of surgery, *n* (%) Open Minimally invasive	62 (16) 318 (84)	24 (10) 215 (90)	0.040
Surgical approach, *n* (%) Transthoracic Transhiatal	310 (82) 70 (18)	172 (72) 67 (28)	0.010
**Procedural information**			
Duration of surgery (hours)	6.4 [5.4–7.4]	6.2 [5–7.2]	0.020
Type of anastomosis, *n* (%) Cervical Intrathoracic	288 (77) 87 (23)	188 (79) 49 (21)	0.530
Any intraoperative complication, *n* (%)	42 (11)	18 (8)	0.150
Conversion to open procedure, *n* (%)	14 (4)	6 (3)	0.570
Estimated blood loss, *n* (%) <500 mL 500–1000 mL >1000 mL	304 (80) 61 (16) 15 (4)	204 (85.5) 34 (14) 1 (0.5)	0.020
At least one unit of RBC needed, *n* (%)	40 (11)	6 (3)	<0.001
Lowest intraoperative pH	7.27 [7.22–7.31]	7.28 [7.23–7.33]	0.060
Intraoperative saturation < 90% for ≥10 minutes, *n* (%)	133 (35)	77 (32)	0.530
On-table extubation, *n* (%)	267 (70)	159 (67)	0.370
**Postoperative course**			
Hospital length of stay (days)	15 [11–24]	12 [10–19]	<0.001
Unplanned ICU (re)admission^†^, *n* (%)	57 (15)	37 (15)	0.960
(Any) Reintubation, *n* (%)	57 (15)	37 (15)	0.960
In-hospital mortality, *n* (%)	18 (5)	6 (3)	0.240

Results are presented as median [IQR] for continuous variables and counts (%) for categorical variables. ^*^Cardiovascular comorbidity is a composite of myocardial infarction, severe coronary artery disease, congestive heart failure, peripheral vascular disease and cerebrovascular disease. ^†^Frequent reasons for ICU readmission (APACHE II diagnoses) included infections (including pneumonia and sepsis) (39%), surgical reasons and/or complications (including anastomotic leakage) (33%) and (noninfectious) respiratory failure (24%). Multiple readmissions were possible for individual patients (totaling 70 and 45 admissions in the ICU-dependent and PACU-eligible groups, respectively). ASA, American Society of Anesthesiology; BMI, body mass index; COPD, chronic obstructive pulmonary disease; eGFR, estimated glomerular filtration rate; FEV1, forced expiratory volume in 1 second; GE gastroesophageal; %pred, percentage of predicted; RBCs, red blood cells.

### Model development

**Fig. 1:**
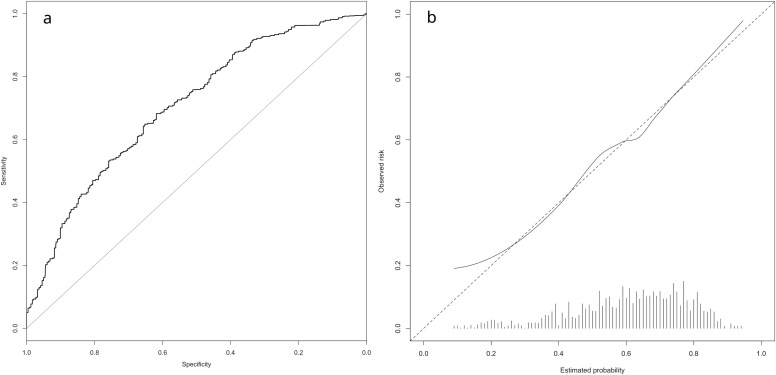
Area under the receiver operating characteristic curve and calibration plot of final model before internal validation. (a) Discriminatory performance (*c*-statistic) of the final model was 0.70 (0.66–0.75), indicating moderate discrimination. (b) Calibration plot of the final model.

After backward selection of candidate predictors, the final model included advanced age, presence of diabetes mellitus, preoperative hemoglobin level < 7.5 mmol/L, lower preoperative forced expiratory volume in 1 second (FEV1), tumor stage ≥ 3, neoadjuvant chemotherapy alone (vs either chemoradiation therapy or no neoadjuvant therapy), open (vs minimally invasive) procedure and transthoracic (vs transhiatal) approach (Table 2). Crude model performance indices indicated moderate discrimination (*c*-statistic 0.70 [95% CI 0.66–0.74]) and good calibration (*O*:*E* ratio 1.00), resulting in fair overall prediction accuracy (rescaled Brier 0.13 [95% CI 0.07–0.18]) (Fig. 1a,b). Subsequent internal validation yielded an adjusted *c*-statistic of 0.67 (95% CI 0.62–0.71), *O*:*E* ratio of 1.00 and rescaled Brier of 0.07 (95% CI 0.02–0.13), again indicating fair model performance overall (Table 3).

**Table 2:** Preoperative prediction model to distinguish patients requiring elective ICU admission from patients that can be cared for in a PACU following esophagectomy

Term	*β*	95% CI	OR	95% CI
Intercept	−1.82	−2.80 to 0.85	0.16	0.06–0.43
Age in years	0.05	0.04–0.07	1.06	1.04–1.07
Diabetes	−0.38	−0.69 to 0.07	0.68	0.50–0.93
Preoperative Hb < 7.5 mmol/L	0.38	0.16–0.59	1.46	1.18–1.81
Preoperative FEV1 (%)	−0.01	−0.02 to −0.007	0.99	0.98–0.99
Tumor stage ≥ 3^*^	0.30	0.09–0.51	1.35	1.09–1.66
Neoadjuvant chemotherapy^†^	−0.94	−1.31 to -0.57	0.39	0.27–0.57
Open procedure^‡^	0.60	0.33–0.88	1.83	1.39–2.41
Transhiatal approach^§^	−0.69	−0.98 to -0.41	0.50	0.38–0.66

^*^T category in TNM classification. ^†^Versus chemoradiation or no neoadjuvant therapy. ^‡^Versus thoracoscopic procedure. ^§^Versus transthoracic approach. FEV1, forced expiratory volume in 1 second.

**Table 3:** Predictive performance estimates after initial model fit (crude) and bootstrap validation (adjusted)

	Crude	Adjusted
C-statistic	0.70 (0.66–0.74)	0.67 (0.62–0.71)
O:E ratio	1.00 (1.00–1.00)	1.00 (1.00–1.00)
Calibration intercept	−0.01 (−0.02 to 0.00)	0.08 (0.07–0.09)
Calibration slope	1.02 (1.00–1.04)	0.81 (0.79–0.83)
Brier	0.21 (0.19–0.22)	0.22 (0.21–0.23)
Rescaled Brier	0.13 (0.07–0.18)	0.07 (0.02–0.13)

Crude estimates correspond to the apparent predictive performance measures after initial model fit. Adjusted estimates show optimism-corrected values obtained by bootstrapping (using 500 iterations).

### Patient disposition

We used a predicted probability cutoff < 0.78 to mark patients for elective admission to the PACU instead of ICU, simulating a 50% reduction in preoperative ICU bed allocations compared to our default strategy (i.e. allocating all patients postoperatively to an ICU). At this decision threshold, the model selected a total of 296 (48%) patients for postoperative recovery at the PACU, of whom 139 (47%, 22% of total cohort) would need to be relocated to the ICU later because they required respiratory and/or cardiovascular support beyond the first morning after surgery. Of note, only 34 (24%) of these patients required continued mechanical ventilation, whereas 68 (49%) merely received low-dose vasopressor support, a therapy that could potentially have been provided at another HDU. Overall, this implies that 25%–42% of ICU beds may be conserved using our model, depending on the local hospital situation (i.e. the various resources available at different wards). A more extensive exploration of various decision thresholds is provided in Supplementary File 4.

### Updated model with intraoperative information

When updating the model with intraoperative information, the model included the same predictors as the preoperative model, but hemoglobin level < 7.5 mmol/L was replaced by intraoperative transfusion. This did not yield improved model performance (adjusted *c*-statistic 0.67 [95% CI 0.63–0.71], *O*:*E* ratio 1.00 [Supplementary File 3 (Table 3, Table 4, Figure 1)]).

## DISCUSSION

At least one-third of patients (and possibly two-thirds if low-dose vasopressor therapy can be delivered elsewhere) do not need admission to an ICU following esophagectomy. Correctly identifying these patients would result in more judicious use of scarce ICU resources. Although our prediction model has only moderate discriminatory performance, it can still decrease elective postoperative ICU bed utilization by approximately 50%, albeit with the consequence of having to reallocate a substantial number of subjects from the PACU to either an HDU or ICU the next day, depending on the local situation.

While advances in perioperative care for esophagectomy patients—such as early extubation and enhanced recovery protocols—have prompted reconsideration of routine ICU admission practices,^7^ it is important to recognize that severe complications typically arise between postoperative days 3 and 10, when patients have usually already been transferred out of the ICU.^3^ Indeed, the results of a large multinational observational study show that routine postoperative ICU admission does not impact postoperative morbidity on day 7 following inpatient surgery.^24^ As a result, current ERAS guidelines recommend individualized postoperative management and suggest that care in a PACU or another HDU may be appropriate for lower risk patients.^7^

Until now, no validated tools have been available to support such risk-based management. Although some prognostic studies have identified predictors such as ASA ≥ 3, a Charlson comorbidity index ≥ 2, absence of neoadjuvant therapy, transthoracic or hybrid surgery, cervical anastomosis and occurrence of intraoperative complications as potential risk factors for prolonged ICU admission (>1 day) in univariable analysis, these were mostly identified through univariate analyses.^15^^,^^24^ In our study, most of these variables were dropped from our multivariable model by the backward selection method used. Moreover, the intraoperative parameters did not convey much predictive information. Instead, our model identified diabetes, preoperative anemia and reduced lung function as key predictors of ICU dependency. These are potentially modifiable risk factors, suggesting that future studies should not only focus on refining prediction tools but also explore whether targeted preoperative optimization could improve postoperative outcomes.

To ensure the universal applicability of our model, we used a strict definition of ICU care that encompassed any level of respiratory and/or hemodynamic support. According to these criteria, 39% of patients were fit for discharge to a regular nursing ward the morning after surgery. However, it is important to note that 28% of all patients merely received infusion of norepinephrine at rates < 0.1 mcg/kg/min. Using our model, we observed that 49% of patients that were misallocated to the PACU also only received low-dose norepinephrine, which may be administered in a variety of HDU settings.

Postoperative care for esophagectomy patients varies significantly across hospitals and countries, including preferences for restrictive versus more liberal fluid administration, HDU availability and protocols for routine ICU stays.^25^ In the Netherlands, e.g., esophagectomy procedures are currently being performed in 15 centers, but there is significant hospital variation in terms of routine postoperative care location. In fact, three hospitals admit esophagectomy patients to a PACU instead of ICU as their standard of care, whereas the others do not.^15^ Both ICUs and PACUs can provide the same supportive care, though patients admitted to the PACU typically must be discharged within 24 hours postsurgery. Patients receiving any low-dose vasopressor support and/or high-flow nasal oxygen after this time can be treated in another HDU when available. For most of the Dutch local settings, the findings of our study therefore imply that potentially a larger proportion of patients than currently estimated by our model could be cared for in a PACU during the first night to 24 hours after surgery, provided that flexible discharge to a HDU is available on the next day. Our model can be adapted to these specific hospital settings by adjusting the probability threshold used to select patients for postoperative disposition to a PACU. Conversely, in facilities that already routinely use PACU admission, the model can be tailored to identify patients who might benefit from ICU care.

Our study has limitations. First, this was a retrospective cohort study, which resulted in some missing data. Since we considered these variables to be missing at random and easy to obtain in clinical practice, we performed multiple imputation by chained equation to impute the missing data. Second, our study cohort spanned over a decade, during which significant advances were made in the care for esophagectomy patients. Importantly, minimally invasive procedures have largely replaced open thoracotomies (from 24% earlier to just 5% in recent years). In fact, adding the calendar year to the model captured essentially the same information as the minimally invasive versus open procedure variable (data not shown), which is a key predictor in our model. As minimally invasive techniques become more standard practice, this reinforces our premise that routine postoperative ICU care can be replaced by PACU admission in many cases. However, we noted an increasing trend in vasopressor dependency over the years, which likely reflects heightened attention to blood pressure management.

Finally, our model was developed based on data from a single center. As discussed above, there is considerable variation in the postoperative care for these patients, both within the Netherlands and internationally. Therefore, this model should be locally validated in these different settings before clinical use.

## CONCLUSION

Over a third of esophagectomy patients do not require routine postoperative ICU admission. An additional third receive only low-dose vasopressor support, suggesting that care outside the ICU may be appropriate in settings with suitable infrastructure. Our prediction model could therefore support more efficient perioperative bed allocation and potentially reduce ICU bed claims by up to 50%.

## Supplementary Material

Supplementary_Materials_finalR2_altered_doaf075

## Notes

Specific author contributions: Nikki de Mul (Conceptualization, Data curation, Formal analysis, Investigation, Methodology, Project administration, Writing—original draft, Writing—review & editing), Lisette Vernooij (Methodology, Supervision, Writing—review & editing), Nynke C. J. van Haastregt (Data curation, Writing—review & editing), Eline Marieke de Groot (Data curation, Writing—review & editing), Willem-Jan M. Schellekens (Conceptualization, Supervision, Writing—review & editing), Lennie P. G. Derde (Supervision, Writing—review & editing), Jelle Ruurda (Conceptualization, Writing—review & editing), Olaf L. Cremer (Conceptualization, Methodology, Supervision, Writing—original draft, Writing—review & editing).

Financial support: None declared.
