# Supplementary material for: Judicious use of critical care resources by predicting the need for routine ICU admission following esophagectomy
Source: Dis Esophagus. 2025 Sep 17;38(5):doaf075. doi: 10.1093/dote/doaf075 (PMC12490070; doi:10.1093/dote/doaf075)
Supplement: Supplementary_Materials_finalR2_altered_doaf075 [file supplementary_materials_finalr2_altered_doaf075.docx]

Supplementary Materials

[1. Candidate predictors 2](#_Toc208404489)

[2. Sample size calculation 3](#_Toc208404490)

[3. Intraoperative model 4](#_Toc208404491)

[4. Selection of the optimal sensitivity-specificity trade-off threshold for our local setting based on predicted probabilities ranging from 0.55 – 0.80 6](#_Toc208404492)

# **Candidate predictors**

| Candidate predictor | Modelled as | Details / Definition | Missing percentage |
| --- | --- | --- | --- |
| *Preoperative predictors* | | | |
| Age | Continuous | Years | 0% |
| Sex at birth | Binary (male / female) |  | 0% |
| Body Mass Index (BMI) | Continuous |  | 0% |
| American Society of Anesthesiologists (ASA) score | Categorized, dummy variables | ASA 3+4 versus ASA1+2 | 1% |
| Cardiovascular comorbidity | Binary (yes/no) |  | 0% |
| Hypertension | Binary (yes/no) |  | 0% |
| Diabetes Mellitus | Binary (yes/no) |  | 0% |
| Smoking (ever) | Binary (yes/no) |  | 0% |
| Scheduled procedure type | Binary | Open versus minimally invasive | 0% |
| Scheduled surgical approach | Binary | Transhiatal versus transthoracic | 0% |
| Tumor T stadium | Categorized | T < 3 versus T ≥ 3 | 0% |
| Neoadjuvant therapy | Categorized, dummy variables | No neoadjuvant therapy versus  Chemotherapy alone versus  Chemoradiation | 1% |
| Estimated glomerular filtration rate (eGFR) | Categorized, dummy variables | eGFR < 60 ml/min versus eGFR ≥ 60 ml/min | 5% |
| Hemoglobin level | Categorized, dummy variables | Hb < 7.5 mmol/L  Hb 7.5 – 8.5 mmol/L  Hb > 8.5 mmol/L | 1% |
| Forced Expiratory Volume in 1 second (FEV1) | Continuous | Percentage of predicted | 6% |
| Tiffeneau index (FEV1/forced vital capacity) | Continuous | Percentage of predicted | 7% |
| *Intraoperative predictors* | | | |
| Duration of surgery | Continuous | Minutes | 0% |
| Intraoperative transfusion | Binary (yes/no) | Any intraoperative transfusion | 0% |
| Any intraoperative complication | Binary (yes/no) | Any intraoperative complications, e.g. conversion, damage to other organs | 0% |
| Intraoperative pH | Continuous |  | 9% |
| Intraoperative hypoxemia | Binary (yes/no) | SaO2 <90% for ≥ 10 min | 0% |
| Type of anastomosis | Binary | Cervical versus intrathoracic | 0% |

**Supplementary Table 1 -** For the preoperative model, only information known prior to surgery was used. Candidate predictors are displayed, including how they were included in the modelling strategy (continuous or categorized) and when applicable, definitions or details of this categorization or predictor are provided. The missing percentages are shown per predictor.

# **Sample size calculation**

| Parameters | C-statistic | Required sample size | With number of events | Events per predictor |
| --- | --- | --- | --- | --- |
| 8 | 0.75 | 366 | 224 | 27.91 |
| 8 | 0.8 | 366 | 224 | 27.91 |
| 8 | 0.85 | 366 | 224 | 27.91 |
| 8 | 0.87 | 366 | 224 | 27.91 |
| 13 | 0.75 | 590 | 360 | 27.68 |
| 13 | 0.8 | 397 | 243 | 18.63 |
| 13 | 0.85 | 366 | 224 | 17.17 |
| 13 | 0.87 | 366 | 224 | 17.17 |
| 18 | 0.75 | 816 | 498 | 27.65 |
| 18 | 0.8 | 549 | 335 | 18.6 |
| 18 | 0.85 | 391 | 239 | 13.25 |
| 18 | 0.87 | 380 | 232 | 12.88 |
| 22 | 0.75 | 998 | 609 | 27.67 |
| 22 | 0.8 | 671 | 410 | 18.6 |
| 22 | 0.85 | 477 | 291 | 13.23 |
| 22 | 0.87 | 464 | 284 | 12.87 |
| 25 | 0.75 | 1134 | 692 | 27.67 |
| 25 | 0.8 | 753 | 466 | 18.62 |
| 25 | 0.85 | 542 | 331 | 13.22 |
| 25 | 0.87 | 527 | 322 | 12.86 |

**Supplementary table 1 – Sample size calculation**

Sample size calculation using the criteria by Riley et al.^[[1]](#footnote-1)^ The only relevant study found in the literature approaching our research question developed a model for predicting prolonged ICU admission after elective noncardiac surgery with a c-statistic of 0.87.^[[2]](#footnote-2)^ When using the criteria by Riley et al., we would use this c-statistic from literature, a (default and recommended) shrinkage factor of 0.90 (correcting for overfitting), and our prevalence of 0.61 for our binary outcome. When making these assumptions, we would be able to include up to 25 candidate predictors (orange). However, as we believed this c-statistic to be optimistic, we also tested other scenarios (varying the number of candidate predictors and the c-statistic) and concluded that 18 candidate predictors would be a more conservative estimate (assuming a somewhat lower c-statistic of 0.80, presented in green).

# **Intraoperative model**

**Supplementary Table 3. Early postoperative model to distinguish patients requiring elective ICU admission from patients that can be cared for in a PACU following esophagectomy**

| **Term** | **β** | **95% CI** | **OR** | **95% CI** |
| --- | --- | --- | --- | --- |
| *Intercept* | -1.74 | -2.71 - -0.76 | 0.18 | 0.07 – 0.47 |
| *Age* | 0.05 | 0.04 – 0.07 | 1.06 | 1.04 – 1.07 |
| *Diabetes* | -0.44 | -0.75 - -0.12 | 0.65 | 0.47 – 0.88 |
| *Preoperative FEV1 (%)* | -0.01 | -0.02 – -0.01 | 0.99 | 0.98 – 0.99 |
| *Tumor T stage ≥ 3* | 0.31 | 0.10 – 0.52 | 1.36 | 1.10 – 1.67 |
| *Neoadjuvant chemotherapy** | -0.99 | -1.36 - -0.61 | 0.37 | 0.26 – 0.54 |
| *Open procedure^#^* | 0.57 | 0.29 – 0.85 | 1.77 | 1.34 – 2.33 |
| *Transhiatal approach^$^* | -0.78 | -1.06 - -0.49 | 0.46 | 0.34 – 0.61 |
| *Transfusion (any)* | 1.57 | 1.23 – 1.91 | 4.79 | 3.42 – 6.72 |

* versus chemoradiation or no neoadjuvant therapy; ^#^ versus thoracoscopic procedure; ^$^ versus transthoracic approach. FEV1: forced expiratory volume in one second.

**Supplementary Table 4. Predictive performance estimates after initial model fit (crude) and bootstrap validation (adjusted)**

|  | **Crude** | **Adjusted** |
| --- | --- | --- |
| *C-statistic* | 0.72 (0.67 – 0.76) | 0.67 (0.63 – 0.71) |
| *O:E ratio* | 1.00 (1.00 – 1.00) | 1.00 (1.00 – 1.00) |
| *Calibration in the large* | -0.01 (-0.13 – 0.11) | 0.08 (-0.04 – 0.21) |
| *Calibration slope* | 1.03 (0.87 – 1.18) | 0.79 (0.64 – 0.94) |
| *Brier score* | 0.20 (0.19 – 0.22) | 0.22 (0.21 – 0.23) |
| *Rescaled Brier score* | 0.14 (0.08 – 0.19) | 0.08 (0.02 – 0.13) |

Crude estimates corresponds to the apparent predictive performance measures after initial model fit. Adjusted estimates show optimism- corrected values obtained by bootstrapping (using 500 iterations).

**Supplementary Figure 1 – Apparent model performance of the postoperative model**


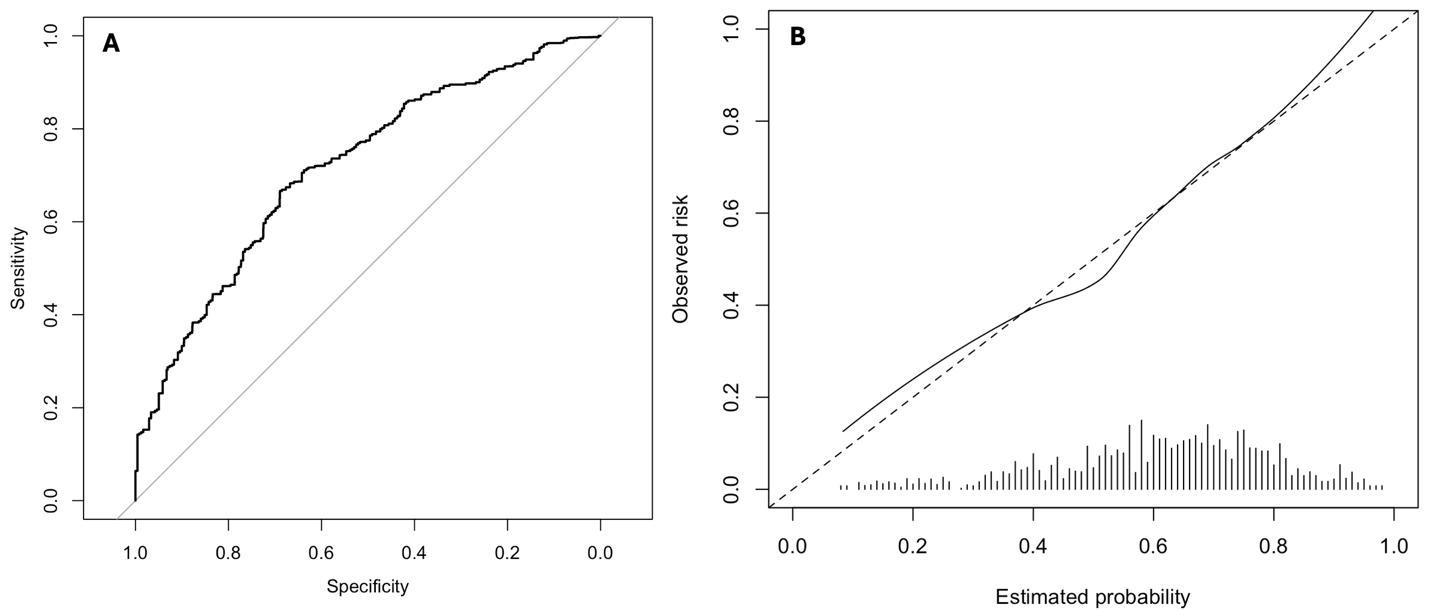


**A.** Discriminatory performance of the postoperative model before internal validation. C-statistic of the final postoperative model was 0.72 (0.67 – 0.76) indicating moderate discrimination. **b.** Calibration of the postoperative model before internal validation.

# **Selection of the optimal sensitivity-specificity trade-off threshold for our local setting based on predicted probabilities ranging from 0.55 – 0.80**

The following tables show predicted and observed patient allocations to an ICU or PACU based on different prespecified thresholds. For example, in the first table, a predicted probability threshold cut-off of 55% is used. This means that a patient is allocated to the ICU when the model predicts a “need for ICU care” of 55% or higher (or allocation to a PACU bed when the model predicts a probability of ICU care <55%). The columns present the observed cases and the rows show the predicted patient flow. Sensitivity, specificity, positive predictive value and negative predictive value are shown below these tables.

Additionally, we illustrate the implications of these numbers for the actual patient flow. The number of ICU beds saved is based on the percentage of patients that are allocated to the PACU based on model predictions (“PACU predicted”), thereby saving ICU beds. The percentage of PACU allocated patients that would actually need ICU care, are patients that are misallocated. This percentage is additionally described based on the total cohort size. The percentage of PACU allocated patients that required mechanical ventilation are presented. This means that at least these patients would have to be relocated to the ICU, making the actual percentage of misallocation smaller as most patients in need of hemodynamic support can be cared for in a high dependency unit (HDU). The percentage of PACU misallocated patients only receiving low dose vasopressors could potentially be discharged to the ward (for example by replacing low-dose vasopressor therapy by fluid administration) or another HDU. The minimal reduction of ICU claims is calculated by subtracting the percentage of misallocation in the total cohort (in absence of a HDU) from the percentage of ICU beds saved (in scheduling).

The table representing the scenario described in the main text is colored orange (predicted probability cut-off 0.78).

Observed patient flow within our cohort (n = 619):

Number of patients requiring ICU care (any support): n = 380 (61%)

Number of patients that are PACU eligible (no support): n = 239 (39%)

Number of patients requiring invasive mechanical ventilation: n = 83 (13%)

Number of patients requiring non-invasive ventilation: n = 28 (5%)

Number of patients requiring inotropes / high-dose vasopressors:* n = 134 (22%)

Number of patients receiving low-dose vasopressors:* n = 204 (33%)

Number of patients *merely* receiving low-dose vasopressors:* n = 174 (28%)

* Vasopressor dosage was stratified into either high-dose (norepinephrine > 0.1 mcg/kg/min or equivalent) or low-dose (norepinephrine ≤ 0.1 mcg/kg/min or equivalent).

1. Riley RD, Snell KI, Ensor J, Burke DL, Harrell FE, Jr., Moons KG, et al. Minimum sample size for developing a multivariable prediction model: PART II - binary and time-to-event outcomes. Stat Med. 2019;38(7):1276-96. [↑](#footnote-ref-1)
2. Lan L, Chen F, Luo J, Li M, Hao X, Hu Y, et al. Prediction of intensive care unit admission (>24h) after surgery in elective noncardiac surgical patients using machine learning algorithms. Digit Health. 2022;8:20552076221110543. [↑](#footnote-ref-2)
